# Evaluating the CURB-65 score for in-hospital mortality prediction in COVID-19 patients: insights into dysglycaemia

**DOI:** 10.1136/bmjph-2024-001291

**Published:** 2024-12-22

**Authors:** Farzahna Mohamed, Brent A Prim, Jarrod Zamparini, Aletta Millen, Frederick Raal, Ismail Sikander Kalla

**Affiliations:** 1 Internal Medicine, University of the Witwatersrand, Johannesburg, South Africa; 2 School of Physiology, University of the Witwatersrand, Johannesburg, South Africa

**Keywords:** COVID-19, SARS-CoV-2, Communicable Disease Control

## Abstract

**Introduction:**

While the CURB-65 score predicts mortality in community-acquired pneumonia (CAP), its performance in COVID-19 CAP is suboptimal. Hyperglycaemia correlates with an increased mortality in COVID-19. This analysis aims to enhance predictive accuracy for in-hospital mortality among COVID-19 patients by augmenting the CURB-65 score with objective variables, including markers of dysglycaemia.

**Design:**

A single-centre retrospective observational analysis assessed the effectiveness of the CURB-65 score in predicting in-hospital mortality among adult patients with moderate to severe COVID-19 from March to September 2020. Using a binary logistic regression model, two extended CURB-65 scores which include markers of dysglycemia are proposed to enhance the predictive capability of the CURB-65 score for in-hospital mortality.

**Results:**

Among 517 patients admitted, 117 (22.6%) died. Using the CURB-65 score, 393 patients (76%) were classified as low risk, 91 (17.6%) as medium risk and 33 (6.4%) as high risk. 37 patients were diagnosed with new-onset dysglycaemia, of which 22 (59.5%) died (p<0.001). Of those with dysglycaemia who died, 41% and 23% were classified as low risk and high risk using the CURB-65 score. The CURB-65 score demonstrated a modest area under the receiver operator characteristic curve (AUC) of 0.75 (95% CI 0.70 to 0.81) for in-hospital mortality in COVID-19 CAP. An Extended CURB-65 Score 1, incorporating an admission of fasting plasma glucose (FPG) and neutrophil to lymphocyte ratio, showed improved prognostic performance with an AUC of 0.80 (95% CI 0.76 to 0.85). When lactate and lactate dehydrogenase were added to these parameters (Extended CURB-65 Score 2), the AUC was 0.82 (95% CI 0.78 to 0.86). The integrated discrimination index showed an 11% and 24% higher discrimination slope when using the Extended CURB-65 Scores 1 and 2, respectively.

**Conclusions:**

The addition of common biochemical parameters including an admission FPG enhances the prognostic performance of CURB-65 for in-hospital mortality among patients with COVID-19.

**WHAT IS ALREADY KNOWN ON THIS TOPIC**

**WHAT THIS STUDY ADDS**

- The inclusion of an admission of fasting plasma glucose to CURB-65 significantly improved predictiveness of in-hospital mortality in COVID-19 patients. This modification offers a cost-effective tool for clinicians in LMICs to better assess mortality risk and optimise care.

**HOW THIS STUDY MIGHT AFFECT RESEARCH, PRACTICE OR POLICY**

- The cytokine storm in SARS-CoV-2 is comparable to that of other viruses, suggesting that COVID-19 mortality prediction models, like the modified CURB-65 with fasting plasma glucose, could be more broadly applied. However, external validation and further research are needed to ensure these models are effectively integrated into clinical practice and policy.

## Introduction

 The global response to the emergence of SARS-CoV-2 has provided valuable insights into managing healthcare crises and the importance of effective triage strategies. In navigating the complexities of the COVID-19 pandemic, healthcare systems have encountered challenges such as overburdened facilities, inadequate resource allocation and the need for timely identification of high-risk patients. The variability in the clinical course of SARS-CoV-2 pneumonia, with patients transitioning from mild symptoms to critical conditions, underscores the necessity for adaptable and cost-effective scoring systems to promptly identify individuals at risk of severe disease.^1 2^

Several scoring systems to identify patients at risk are already well established. Over two decades ago, the British Thoracic Society recommended the use of the CURB-65 score, which includes mental confusion, urea, respiratory rate, blood pressure and age ≥65 years for managing community-acquired pneumonia (CAP).^3^ The Pneumonia Severity Index is another clinical predictive score that categorises patients based on 20 clinical and laboratory parameters, displaying strong discriminatory ability.^4^ However, its complexity in the calculation, especially at the time of hospital admission, led to the preference for the simpler CURB-65 score. In South Africa, where CURB-65 is recommended for CAP, its application in COVID-19 has revealed higher mortality rates in low-risk groups, emphasising the need for region-specific research to improve predictive tools.^5^ Data from the primary analysis of our study, evaluating the performance of severity scores and risk factors for in-hospital mortality, showed that the National Early Warning Score 2 (NEWS2) and the International Severe Acute Respiratory and Emerging Infections Consortium- Coronavirus Clinical Characterisation Consortium Mortality Score (ISARIC-4C) performed better than the CURB-65.^5^ This suggests that the CURB-65 score has unexplained risk factors that are not accounted for, thereby constraining its effectiveness in predicting mortality in COVID-19. Recommendations for managing CAP suggest home-based care for low-risk groups identified by the CURB-65 score with a predicted mortality rate below 4%.^5^ While commonly used severity scores like CURB-65 may lack specificity for COVID-19, the incorporation of additional objective biochemical variables has the potential to enhance predictive accuracy.^2 3 6^ An extended CURB-65 was recommended for CAP patients due to its accuracy and simplicity.^6^ Several studies suggest improving CURB-65 by adding biochemical factors, such as D-dimer and procalcitonin, to predict mortality in hospitalised SARS-CoV-2 CAP.^7^ However, these additions did not significantly enhance the effectiveness of CURB-65 in predicting critical outcomes. Conversely, one study illustrates that incorporating hypoalbuminaemia (albumin <3.5 g/dL) boosts the predictive accuracy for in-hospital mortality.^2^ We propose that a glucocentric approach is essential in triage scores like CURB-65 due to the significant impact of hyperglycaemia (HG), on mortality rates in severe infections like CAP. HG, even without diabetes mellitus (DM), is associated with higher mortality rates and worse outcomes than DM alone.^8^,^10^ New-onset HG and elevated fasting plasma glucose (FPG) on admission are independent risk factors for mortality in COVID-19, contributing to higher mortality rates and longer hospital stays, as supported by other research.^8 9 11 12^ Incorporating glucose parameters into triage scores will allow for an immediate and easily accessible mortality risk assessment enabling quick mortality risk prediction for timely and appropriate management decisions. By leveraging these insights, we seek to enhance the accuracy and prognostic value of triage systems, ultimately improving patient outcomes in future healthcare crises.

This study’s primary objective was to evaluate the accuracy and prognostic value of adding biochemical markers of dysglycaemia, including an FPG on admission to the CURB-65 score, specifically in predicting in-hospital mortality in patients with SARS-CoV-2-associated CAP.

## Research design and methods

### Study setting

This single-centre study was conducted at the Charlotte Maxeke Johannesburg Academic Hospital, a tertiary state hospital in South Africa, from March to September 2020, and retrospectively analysed 517 adults through a single-centre cohort design. The inclusion criteria involved adult patients, ≥18 years of age, admitted with laboratory-confirmed positive SARS-CoV-2 reverse transcriptase PCR from oropharyngeal or nasopharyngeal swabs, with moderate to severe COVID-19 symptoms. The predominant variant during this period was the ancestral variant with the Asp614Gly mutation.^13^ Patients were excluded if they had mild COVID-19 that did not require inpatient admission, as well as pregnant women.

### Data collection

Relevant demographic and clinical data were anonymously extracted from a secure electronic database. Essential parameters for calculating the CURB-65 score, including age (years), mental state, urea (mmol/L), respiratory rate (breaths/min) and systolic and diastolic blood pressure, were captured. Patients were categorised into three risk groups: low, medium and high risk, based on aggregate scores (online supplemental table 1).

### Definitions

Pneumonia was defined in accordance with the WHO interim guidance for the clinical management of COVID-19, characterised by clinical signs of pneumonia such as fever, cough, dyspnoea and fast breathing for <2 weeks. Severity was categorised as moderate when the oxygen saturation (SpO2) was >90%, without signs of severe pneumonia such as respiratory rate >30 breaths/min, severe respiratory distress or SpO2 <90% on room air.^14^ DM was defined as glycated haemoglobin (HbA1c) ≥6.5% during the admission or as having pre-existing DM. Uncontrolled HG was defined as two or more blood glucose recordings>10 mmol/L within 24 hours with an HbA1c ≤ 6.5% or if an HbA1c was not done.

### Statistical analysis

Data analysis was performed by using RStudio and R Statistical Software. A Mann-Whitney U test was used to compare quantitative variables between those who survived vs those who did not. Pearson’s χ^2^ test and Fisher’s exact (two-tailed) test were used to analyse qualitative data. A multivariate imputation technique was used to impute the missing values for the FPG, lactate on arterial blood gas (ABG), lactate dehydrogenase (LDH) and troponin (online supplemental figure 1). The imputed values shared the same distribution as the observed data. Identification of risk factors for in-hospital mortality was conducted through univariate logistic regression. All variables with p≤0.25 in the univariate analysis were considered candidate variables for the multivariable analysis. The optimal cut point for each predictor was determined using receiver operator characteristic curve (ROC) analysis and a backward selection method was used to select the driving factors among low-risk patients who experienced mortality (online supplemental figure 2). Following the result from the multivariable logistic regression analysis, an extended CURB-65 score was determined by adding each predictor step by step. Each patient was risk stratified based on CURB-65. Risk groups were defined based on established cut-off values (online supplemental table 1).^5^ The performance of CURB-65 for in-hospital mortality was assessed through ROC analysis.

A binary logistic regression model was proposed to identify the driving factors in the low-risk CURB-65 group and two extended CURB-65 scores were proposed. Optimal cut points for each predictor were determined using ROC analysis. Predictors were then selected based on clinical relevance and cost. The first extended score is a cost-effective score based on additive variables that are easily accessible in a resource-limited setting to enhance early decision-making. The second extended score depends on further laboratory analysis that may not be accessible in all settings but may be associated with a delay in implementing the score. The variables that are included in the extended CURB-65 scores were selected based on the area under the curve (AUC), sensitivity, specificity, positive predictive value (PPV), negative predictive value (NPV) and integrated discrimination index (IDI). The IDI is often used when comparing a new model that includes additional predictors against a reference model, therefore, the CURB-65 score was used as a reference to calculate the IDIs for each extended CURB-65 score under the following hypothesis:

- H0: The addition of new predictors to the existing CURB-65 score does not improve the model’s ability to discriminate between survival (alive) and mortality (dead) outcomes in COVID-19 patients.
- H1: The addition of new predictors to the existing CURB-65 score does improve the model’s ability to discriminate between survival (alive) and mortality (dead) outcomes in COVID-19 patients.

The Hosmer and Lemeshow test was used to assess how well the multivariable logistic regression model fits the data, with a p>0.05 showing a good fit as it fails to reject the null hypothesis.

We incorporated a 5-fold and 10-fold cross-validation to assess the performance of the CURB-65 score and the Extended CURB-65 Scores (score 1 and score 2) using Cohen’s kappa coefficient. This method was applied to evaluate the agreement between predicted and observed outcomes, accounting for classification consistency beyond chance (online supplemental table 2).

### Patient and public involvement

Patients and/or the public were not involved in the design, conduct, reporting or dissemination plans of this research.

## Results

### Study cohort

Of the 517 patients included in the study, 117 (22.6%) died during the study period of which 45 (38.5%) were female (table 1). Using the original CURB-65 score, 393 patients (76%) were identified as low-risk group, whereas 91 (17.6%) and 33 (6.4%) were considered medium and high risk, respectively (table 1). There was a significant association between the risk groups and in-hospital mortality (p<0.001) (table 1). Among the total, 134 (25.9%) had DM and 37 patients were diagnosed with new-onset dysglycaemia, of which 22 (59.5%) died. Of those with new-onset dyslglycaemia who died, 9 (40.9%), 8 (36.4%) and 5 (22.7%) were categorised as low, medium and high risk as per CURB-65, respectively. There was a significant association between new-onset dysglycaemia and in-hospital mortality (p<0.001). When comparing the differences in demographic and biochemical variables between survivors and non-survivors, non-survivors were significantly older than survivors with a mean age of 57 and 51 years, respectively. The differences in the biochemical variables between those who survived versus those who did not are shown in table 2.

**Table 1:** Association of sex, CURB-65 risk and glycaemic status with in-hospital mortality

Characteristics	Overall	Survived	Non-survived	COR	95% CI	P value*
	n (%)	n (%)	n (%)			
N	517	400 (77.4)	117 (22.6)			
Sex						
Male	270 (52.2)	198 (49.5)	72 (61.5)	Ref		0.027
Female	247 (47.8)	202 (50.5)	45 (38.5)	0.61	0.39, 0.95	
Risk group by CURB-65 score						
Low risk	394 (76.2)	341 (85.3)	53 (45.3)	Ref		*<*0.001
Medium risk	88 (17)	46 (11.5)	42 (35.9)	5.75	3.48, 9.51	
High risk	35 (6.8)	13 (3.2)	22 (18.8)	11.23	5.22 24.15	
New diabetes						
No	388 (75)	306 (76.5)	82 (21.1)	Ref		0.158
Yes	129 (25)	94 (23.5)	35 (27.1)	0.72	0.46, 1.14	
New-onset dysglycaemia						
No	480 (92.8)	385 (96.3)	95 (19.8)	Ref		*<*0.001
Yes	37 (7.2)	15 (3.75)	22 (59.5)	0.17	0.08, 0.34	

Results are presented as frequencies and percentages for qualitative variables. Pearson’s χ2 test and Fisher’s exact (two-tailed) test were used to analyse qualitative data.

* χ2. Statistical significance p≤0.05.

CORcrude OR

**Table 2:** Differences in biochemical variables between survivors versus non-survivors

Characteristics	Overall	Survived	Non-survived	P value
N		400	117	
WCC (×10^9^/L)	8 (6, 11)	7 (5, 10)	10 (7, 14.5)	*<*0.001
Neutrophils (×10^9^/L)	6 (4, 9)	5 (4, 8)	8 (5, 12)	0.001
Lymphocytes (×10^9^/L)	1 (1, 2)	1 (1, 2)	1 (1, 1.5)	0.452
NLR (×10^9^/L)	5 (3, 9)	4 (3, 7)	8 (5, 13)	0.001
CRP (mg/L)	91 (36, 1911)	78.5 (30,166)	165 (73.5, 270)	*<*0.001
Ferritin (μg/L)	635 (289, 1448)	553 (238.8, 1341)	904 (473, 1907)	0.104
LDH (U/L)	482 (349, 663)	434.5 (324, 631)	638 (463, 813)	*<*0.001
Lactate on ABG (mmol/L)	2 (1,2)	2 (1, 2)	2.9 (1, 3)	0.001
AST (U/L)	43 (29, 63)	41 (28, 58.8)	51 (37, 84)	*<*0.001
ALT (U/L)	28 (19, 45)	27.5 (19, 45)	28 (18, 50)	0.313
D-dimer (mg/L)	1 (0,2)	1 (0, 2)	1 (1, 2)	0.004
Troponin (ng/L)	12 (5.30)	10 (5, 28.8)	22 (9, 69.5)	0.019
FPG (mmol/L)	9 (6, 14)	8 (6, 13)	11 (7, 16)	*<*0.001

Results are presented as median (IQR) and the Mann-Whitney U test is used for statistical comparison. Statistical significance p≤0.05.

ABGarterial blood gasALTalanine transaminaseASTaspartate transaminaseCRPC reactive proteinFPGfasting plasma glucoseLDHlactate dehydrogenaseNLRneutrophil to lymphocyte ratioWCCwhite cell count

### Performance of CURB-65 and predictors of in-hospital mortality in the low-risk group

**Figure 1:**
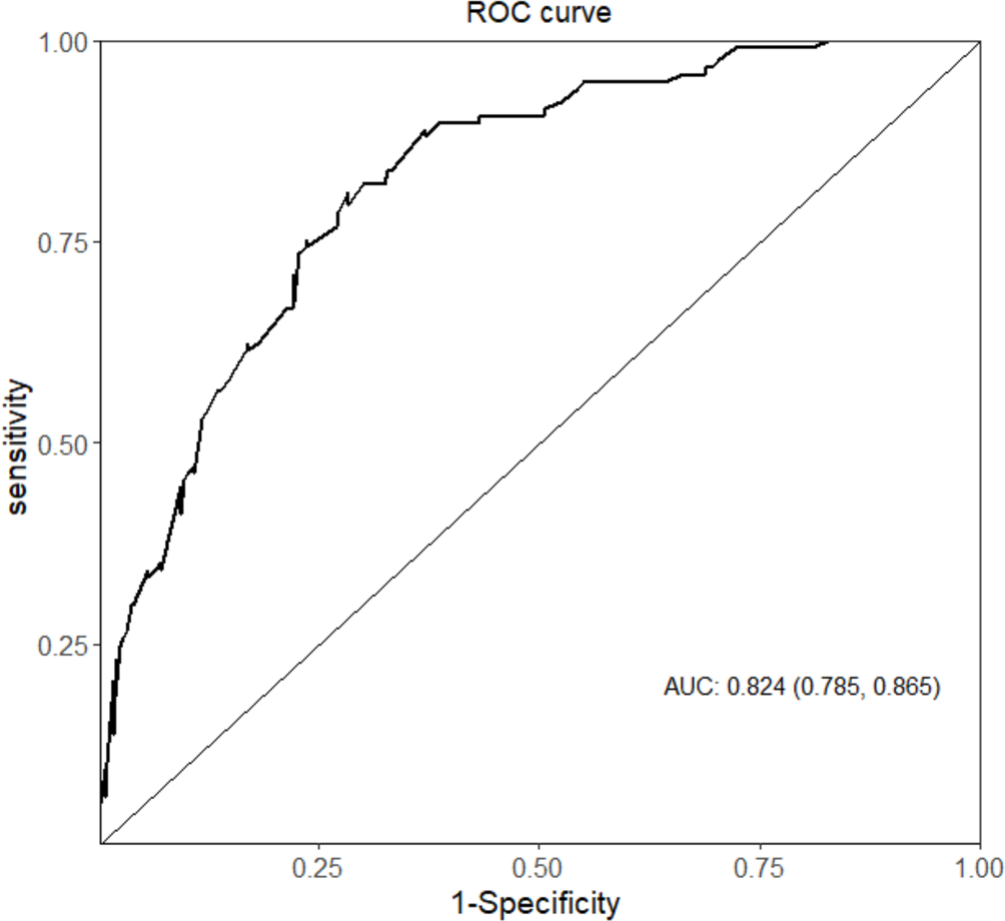
ROC analysis for CURB-65 in the low-risk category. AUC, area under the curve; ROC, receiver operator characteristic.

A higher mortality rate was observed in each category of the CURB-65 score (table 1). The distribution of patients in the overall cohort (n=517) by CURB-65 and extended CURB-65 categories is shown in online supplemental figure 3. A 13%, 47% and 63% overall mortality rate occurred within the low-risk, medium-risk and high-risk groups, respectively (online supplemental figure 4). However, 45% of the overall patients who died were classified as low risk (online supplemental figure 5). The overall mortality rate for each score, within each point category of the score, is shown in online supplemental figure 5. The AUC for those with low-risk CURB-65 in predicting in-hospital mortality was 0.824 (95% CI 0.785 to 0.865) in patients with SARS-CoV-2 CAP (figure 1). Multivariable regression analysis for in-hospital mortality of patients within the low-risk CURB-65 category was performed to identify other factors that are independently associated with mortality (table 3). Laboratory parameters that are not included in the CURB-65 score, but that significantly predicted in-hospital mortality include an admission FPG, lactate on ABG, LDH, aspartate transaminase (AST) and neutrophil to lymphocyte ratio (NLR) (table 3).

**Table 3:** Multivariable regression analysis for in-hospital mortality of low-risk COVID-19 patients as per CURB-65 score

Predictor	AOR	95% CI	P value
FPG <9.3	(Reference)	--	
FPG ≥9.3	1.98	1.04, 3.88	0.040
ABG lactate <2.3	(Reference)	--	
ABG lactate ≥2.3	2.28	1.10, 4.63	0.023
LDH <449	(Reference)	--	
LDH ≥449	2.32	1.15, 4.94	0.022
AST <57	(Reference)	--	
AST ≥57	2.63	1.39, 5.02	0.003
NLR <4.35	(Reference)	--	
NLR ≥4.35	4.78	2.19, 11.63	<0.001

Statistical significance p≤0.05.

ABGarterial blood gasAORadjusted ORASTaspartate transaminaseFPGfasting plasma glucoseLDHlactate dehydrogenaseNLRneutrophil to lymphocyte ratio

**Figure 2:**
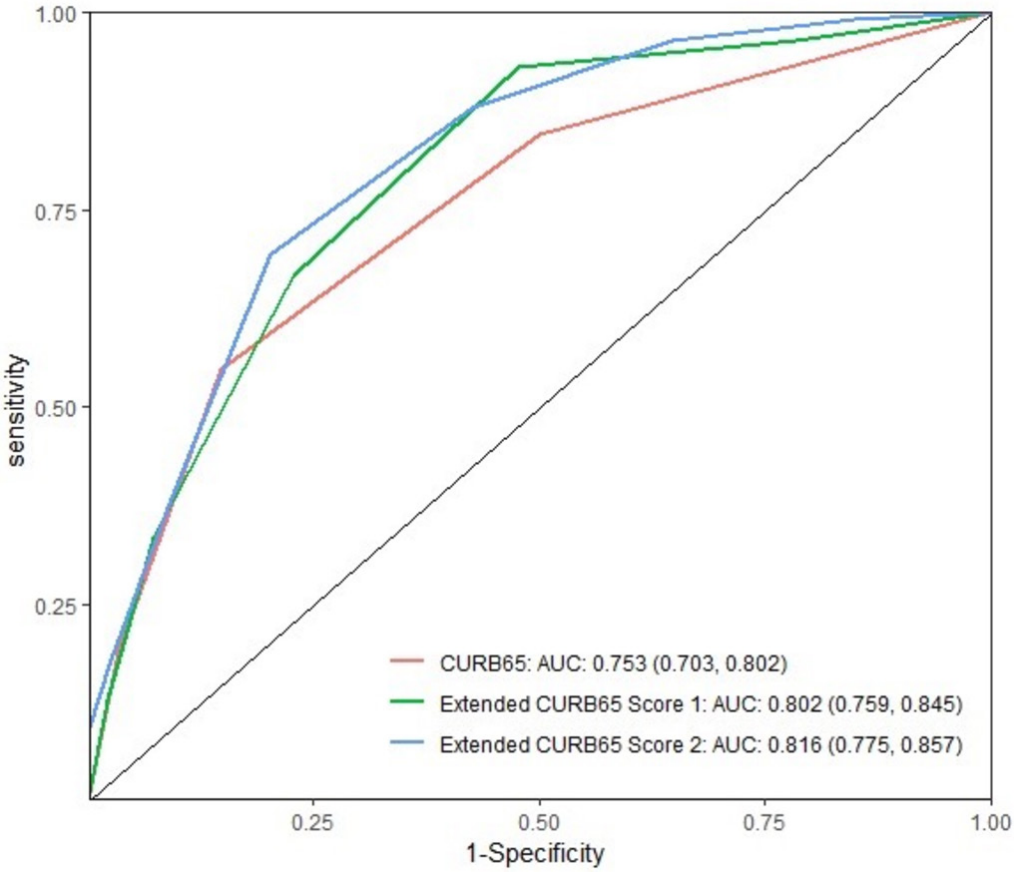
ROC analysis for the Extended CURB-65 Scores to predict in-hospital mortality. AUC, area under the curve; ROC, receiver operator characteristic.

Based on these findings, these additional five laboratory parameters were then included at optimal cut-off values in the CURB-65 score. ROC curve analysis evaluated the prognostic ability of including the additional variables to the standard CURB-65 score in predicting in-hospital mortality and showed improved performance when ≥2 additional laboratory parameters were added (online supplemental table 3, online supplemental figure 2). The first proposed extended score includes parameters that are readily available on admission, including, NLR and FPG (online supplemental table 3). The second extended score includes FPG, NLR, lactate on ABG and LDH (online supplemental table 3). Online supplemental table 4 shows the sensitivity, specificity, PPV and NPV for the two extended scores. A statistically significant improvement was found with both proposed Extended CURB-65 Scores as compared with using CURB-65 independently, with an integrated discrimination improvement of 0.116 (95% CI 0.107 to 0.125) and 0.235 (95% CI 0.224 to 0.245), respectively (online supplemental table 3). The ROC curves comparing the AUC for the CURB-65 score to the two extended scores are shown in figure 2. The risk of mortality at a cut-off of <2 and <4 was 1.54% and 6.96% for the Extended CURB-65 Score 1 and score 2, respectively (online supplemental tables 4–6). The Hosmer and Lemeshow test indicated a good fit of the data (χ^2^=6.27, p=0.098).

## Discussion

The study highlighted the limitations of using the CURB-65 score for predicting in-hospital mortality in SARS-CoV-2-associated CAP. It revealed a 13.5% mortality rate in the low-risk CURB-65 group, with 45% of all deaths occurring in this group. We identified five independent risk factors for mortality in low-risk CURB-65 patients with SARS-CoV-2, including admission FPG, lactate on ABG, LDH, AST and NLR. We proposed two extended CURB-65 scores based on statistical performance and clinical applicability. The Extended CURB-65 Score 1 serves as a cost-effective tool that can be easily implemented at most healthcare facilities regardless of resource limitations and includes an FPG and NLR in addition to the CURB-65 score. In contrast, Extended CURB-65 score 2 incorporates additional predictors to the first score, including LDH and lactate on an ABG, that are more costly and are not readily available, potentially delaying the severity assessment. The study showed improved prognostic performance, with both extended scores demonstrating a higher AUC and NPV compared with the original CURB-65 score. A substantial improvement in the discrimination slope when using the extended CURB-65 scores was also demonstrated. The substantial variation between the PPV and NPV observed is likely due to the higher incidence of adverse outcomes with increased mortality in those with SARS-CoV-2 CAP within the cohort. Despite this, a consistently high NPV exceeding 90% with each additional variable added to CURB-65, and an NPV of 94% and 92% in Extended Scores 1 and 2, respectively, highlight their effectiveness in identifying patients at low risk for mortality, emphasising their optimal clinical utility in this regard.

In those with low-risk CURB-65 within our study, an admission FPG ≥9.3 mmol/L was associated with 1.98 increased odds of in-hospital mortality. Data from our centre within the same COVID-19 wave showed that individuals with HG experienced higher mortality, increased ventilation needs and intensive care unit (ICU) admission, with a 3.3-fold increase in mortality, ventilation rates and a 3.5-fold greater likelihood of ICU admission on univariate analysis.^8^ Multivariate analysis confirmed HG as an independent risk factor for COVID-19 mortality, irrespective of diabetic status, aligning with international findings linking increased admission FPG and glucose fluctuations to increased mortality and prolonged hospital stays.^8^,^1015^ Studies have shown that even a slight increase in FPG within the normal range poses a substantial risk of increased mortality, with a 1 mmol/L increase in FPG corresponding to 1.59 times higher odds of ICU admission, translating to a significant 10.45 times higher odds for every 5 mmol/L increase.^19^ FPG has also been shown to be associated with an increase in hospitalised mortality rates and poor early outcomes in a J-shaped manner, and a combination of HG, inflammation, hypercoagulation and cytokines conferred a dramatically higher risk.^20^

The precise mechanisms for new-onset diabetes and HG in people with COVID-19 are uncertain. Profound changes in overall metabolism and persistent inflammation impacting the body’s glucose balance have been proposed as potential mechanisms of HG.^17^ The immune system’s activation during viral infection leads to systemic insulin resistance.^17^ An elevated triglyceride and glucose (TyG) index was shown to be an independent predictor of severe disease and increased mortality in COVID-19, supporting the possibility of insulin resistance contributing to HG in infected individuals.^21^ Heightened expression of angiotensin-converting enzyme 2 (ACE2) receptor and potential pancreatic injury may contribute to HG in COVID-19 patients with DM.^17^ However, data on the association of ACE2 expression in islet cells with COVID-19 severity are lacking. Additionally, stress-related HG, steroid-induced effects and the potential impact on β-cells by the virus have also been postulated as contributory factors.^18^ Recognising these links is essential for clinicians to effectively handle COVID-19 cases, underscoring the significance of monitoring blood glucose levels.^17^ Urgent research is needed to understand and manage these complexities, especially given the increased mortality in cases of new-onset DM. Hospitals should incorporate protocols for recognising and managing acute HG. The long-term persistence of new-onset DM remains uncertain, requiring prospective studies for a comprehensive understanding. Hence, recognising moderately elevated FPG is crucial for predicting ICU admission risk, aiding clinicians in managing epidemics.^19^ Research conducted during the same wave of COVID-19 at our centre revealed significantly higher inflammatory markers including CRP, LDH and ferritin in individuals with DM and HG compared with those with euglycaemia (EG), in predicting ICU admission, need for invasive ventilation and in-hospital mortality. However, only an elevated level of LDH emerged as a significant predictor of mortality, ICU admission and the need for ventilation, with an LDH level exceeding 350 U/L being associated with 3.5 times greater odds of mortality.^5^

LDH, an intracellular enzyme present in various organs and tissues, is released during cell injury or death caused by factors like ischaemia, bacterial toxins, extreme temperatures, starvation, dehydration, injury, drugs and chemical poisonings.^6 22 23^ The heightened LDH levels indicate insufficient tissue perfusion, and its concentrated presence in different organs means even minor injuries can significantly raise serum LDH levels.^6^ Previous studies underscore its utility in evaluating lung tissue damage and inflammation in various conditions including pulmonary embolism, *Pneumocystis carinii* pneumonia, tuberculosis, bacterial pneumonia, influenza A and COVID-19.^6 24 25^ Elevated LDH in patients with COVID-19 signals lung and tissue injuries, potentially leading to inadequate tissue perfusion and multiple organ failure, often involving thrombosis that causes LDH elevation.^26^ Consequently, elevated LDH serves as a biomarker for disease severity. Our current analysis showed that an LDH >449 U/L in those categorised within the low-risk CURB-65 group had 2.32 times greater odds of in-hospital mortality. Despite some studies showing an independent association between elevated LDH and a poor prognosis, especially when LDH exceeds >250 U/L, pooled results reveal that LDH exhibits poor predictive performance when used alone.^26^ This may be due to the heterogeneity of diagnostic assays and cut-off levels in various studies. Hence, it warrants further investigation and integration into a comprehensive risk prediction model alongside another biochemical marker.^26^ The incidence of an elevated LDH has also been shown to be linked to DM, possibly stemming from diminished glycogen synthesis, alterations in glucose oxidative metabolism, and an increased overall rate of non-oxidative glycolysis.^26^ These mechanisms are thought to cause an elevated lactate in patients with insulin resistance compared with those without.^26^,^28^

Our study showed that in those with low-risk CURB-65, a lactate >2.3 mmol/L on an ABG was associated with 2.28 increased odds of in-hospital mortality. A systematic review analysis of 19 studies involving 6459 patients evaluated the potential correlation between elevated blood lactate levels and disease severity and/or mortality in COVID-19.^29^ The findings suggested that individuals with worse outcomes generally exhibited higher lactate values, proposing that monitoring blood lactate during hospitalisation could facilitate the early identification of a higher risk of unfavourable COVID-19 progression.^29^ While elevated lactate is frequent in patients with COVID-19 and unfavourable clinical progression, the levels observed are not consistently as high as in severe non-COVID-19 pneumonia, acute respiratory distress syndrome or sepsis, urging caution in applying traditional thresholds. Studies suggest frank hyperlactataemia >2.0 mmol/L or even 3 mmol/L typical of severe non-COVID-19 pneumonia or sepsis is mainly present before death in deceased COVID-19 patients, underscoring the importance of a nuanced interpretation of lactate values in this context.^29^ Lactate is an important metabolite in the glycolytic pathway, and research has shown that muscle lactate/pyruvate metabolism is altered in obese, non-insulin-dependent DM patients with fasting HG thereby impacting insulin sensitivity.^26^,^28^ A progressive and proportional increase in plasma lactate levels has been shown in oral glucose tolerance test, hyperinsulinaemic-euglycaemic clamp studies, as well as epidemiological studies, implying that lactate may serve as an early marker of insulin resistance long before a diagnosis of DM, especially in those with a BMI>30 kg/m^2^.^26 30 31^ Prolonged hyperlactataemia, fueled by increased lactate production from adipocytes in obese individuals, occurs before the onset of DM and may contribute to its development. However, the exact molecular mechanisms for the association of lactate to insulin resistance and DM remain unclear, but it is suggested that processes such as inhibiting glucose oxidation, suppressing glucose transport, impeding insulin-stimulated glycolysis and reducing insulin-induced glucose uptake may be involved.^26 27^ Additionally, lactate-induced insulin resistance is thought to be linked to compromised insulin signalling and diminished insulin-triggered glucose transport in skeletal muscle.^26 28 30 31^

Finally, the addition of NLR to the CURB-65 score significantly improved its performance. Elevated NLR is linked to increased mortality and disease severity in COVID-19 patients.^32^,^35^ The cut-off values for prognosis vary among studies, with our study showing 4.78 times increased risk of mortality at a cut-off >4.35. Studies have also highlighted that the NLR is an independent risk factor for critical illness and hospital mortality in COVID-19 patients, whereby patients aged ≥50, with an NLR ≥3.13 were predicted to develop critical illness.^36^ Despite a lack of consensus on the optimal NLR value, a systematic analysis of over 90 studies, with over 13 000 patients with COVID-19, reveals that higher NLR on admission correlates with poor outcomes, whereby the ROC for mortality and severity showed an AUC of 0.87 and 0.82, respectively.^35^ The elevation of the NLR in COVID-19 is attributed to the virus’s association with lymphopenia, which is common in viral infections.^32^ Additionally, neutrophils from COVID-19 patients induced T-cell polarisation, leading to a reduction in the percentage of T1 cells. NLR is thus a reflection of the systemic inflammatory status and is easily obtained from the differential white cell count, serving as a predictive biomarker for mortality even in the general population.^32^ This highlights the potential significance of the NLR in predicting mortality and disease severity and holds promise as a prognostic tool in COVID-19, aiding risk stratification and resource optimisation.

However, not all biomarkers were of clinical relevance. Our study showed that an AST>57 U/L (1.6×the upper limit of normal) was associated with 2.63 odds of in-hospital mortality in COVID-19. AST was excluded from the proposed COVID-19 extended CURB-65 score due to uncertainties surrounding the significance of abnormal liver biochemistries in SARS-CoV-2 patients. COVID-19 often leads to elevated liver enzymes, including AST, with various potential causes such as direct liver injury, inflammation, hepatopathy, ischaemia, drug-induced injury and muscle breakdown.^37 38^ Bloom *et al* highlighted diagnostic challenges in assessing elevated liver enzymes in COVID-19 patients, showing low agreement among hepatologists on the most likely diagnosis.^38^ Current recommendations from the gastroenterology and hepatology societies endorse a thorough evaluation and monitoring for alternative causes of elevated liver enzymes in COVID-19 patients.^39 40^ This was based on a metanalysis of 47 studies, analysing over 10 000 patients with COVID-19, showing a pooled prevalence of elevated AST of 15.0% and alanine transaminase of 15.0%, underlining the importance of comprehensive assessment and monitoring.^39^

This study has certain limitations, stemming from its retrospective, single-centre design that excluded outpatients with mild disease. The absence of predefined prognostic cut-off values for proposed biochemical parameters in SARS-CoV-2 CAP patients prompted the use of a ROC curve to optimise sensitivity and specificity. The study’s small sample size at a single centre poses external validity challenges and potential biases in both the proposed and reviewed models. The multivariate analysis was performed to identify biomarkers in addition to the CURB-65 score that will improve its performance in the low-risk group, however, the proposed extended scores are intended for use and validation in all those admitted with COVID-19. Additionally, the findings may not be widely applicable due to the heterogeneity of COVID-19 disease phenotypes resulting from viral mutagenicity with each COVID-19 wave. The study predates widespread vaccination, and the impact of vaccination on current predictive severity scoring systems requires further research, particularly in vaccinated cohorts. The study’s strength lies in it being the first to evaluate the accuracy of an extended CURB-65 score using glucose as a biomarker in predicting mortality. Our findings contribute to the existing literature confirming the association between elevated FPG, LDH, lactate and NLR with adverse outcomes, though its discriminatory ability requires additional investigation. Although the suggested extended CURB-65 score introduces a more straightforward tool for assessing the severity of hospitalised patients with CAP, the study emphasises the need for validation in a prospective, multicentre cohort with diverse demographics and resources for robustness. Despite the absence of an external dataset and the uncertainty of validating the model in a future COVID-19 wave, we hope this study inspires future validation efforts in other viral-mediated pandemics, as the cytokine storm observed in SARS-CoV-2 is comparable to that of other viruses. Data on the association of mortality and new-onset diabetes with body mass index, inflammatory markers representative of the TH1, TH2 and TH17 pathways, as well as markers of insulin resistance including the TyG index, are currently being analysed within a prospective cohort.

## Conclusion

In resource-constrained environments, the timely assessment of mortality risk is crucial for healthcare professionals to prioritise and enhance care for high-risk patients. The introduction of the two extended CURB-65 scores offers a streamlined and effective approach to assessing the severity of hospitalised patients with SARS-CoV-2 CAP. The proposed score should be used to guide but not replace clinical decision-making. While the initial results show promise, further validation is warranted through subsequent large-scale, multicentre prospective studies across diverse patient populations.

## supplementary material

10.1136/bmjph-2024-001291 online supplemental material 1

## Data Availability

Data may be obtained from a third party and are not publicly available.
