# Supplementary material for: Evaluating the CURB-65 score for in-hospital mortality prediction in COVID-19 patients: insights into dysglycaemia
Source: BMJ Public Health. 2024 Dec 22;2(2):e001291. doi: 10.1136/bmjph-2024-001291 (PMC11816281; doi:10.1136/bmjph-2024-001291)
Supplement: online supplemental material 1 [file bmjph-2-2-s001.pdf]

Supplementary Appendix

List of supplementary tables

Table 1: CURB-65 scoring and risk categorization.

Table 2: Agreement of the Extended CURB-65 Scores with CURB-65.

Table 3: Logistic regression model with ROC analysis in the low-risk CURB-65 patients.

Table 4: ROC analysis for the Extended CURB-65 Scores predicting in-hospital mortality in the low-risk CURB-65.

Table 5: Extended CURB-65 Score 1 with mortality risk as per risk category.

Table 6: Extended CURB-65 Score 2 with mortality risk as per risk category.

List of supplemental figures

Figure 1: Density plot comparing the distribution of observed and imputed values.

Figure 2: ROC analysis for FPG, lactate on ABG, AST, LDH, NLR for COVID-19 patients.

Figure 3: The distribution of patients in the overall cohort by CURB-65 and Extended CURB-65 categories.

Figure 4: The distribution of mortality in each risk category of CURB-65 and Extended CURB-65 Scores.

Figure 5: The distribution of mortality in the overall non-survival cohort by CURB-65 and Extended CURB-65 categories.

Table 1 CURB-65 scoring and risk categorization. [5]

CURB-65 Score		
**Parameter**	**Threshold**	**Points**
Mental state	Confusion	1
Urea (mg/L)	≥ 7	1
Respiratory rate (breaths/min)	≥ 30	1
Blood pressure (mmHg)	Systolic < 90 or Diastolic < 60	1
Age (years)	≥ 65 years	1
**Aggregate Score**	**Risk**	**Mortality (%)**
0-1	Low risk	0-4
2	Moderate risk	17
≥ 3	High risk	26-33

[5]Prim BTA, Kalla IS, Zamparini J, et al. COVID-19: An evaluation of predictive scoring systems in South Africa. Heliyon 2023;9:e21733. https://doi.org/10.1016/j.heliyon.2023.e21733.

Table 2 Agreement of the Extended CURB-65 Scores with CURB-65.

Scores	5-fold cross validation		10-fold cross validation	
	**Accuracy**	**Kappa**	**Accuracy**	**Kappa**
CURB-65 score	0.790	0.207	0.791	0.205
Extended CURB65 Score 1	0.791	0.296	0.793	0.306
Extended CURB65 Score 2	0.795	0.304	0.798	0.312

Table 3 Logistic regression model with ROC analysis in the low-risk CURB-65 patients.

**CURB-65 + variable**	**AUC (95% CI)**	**Sensitivity**	**Specificity**	**PPV**	**NPV**	**IDI (95% CI)**
CURB-65	0.753 (0.703, 0.802)	0.547	0.852	0.520	0.865	
CURB-65 + FPG	0.776 (0.729, 0.823)	0.735	0.685	0.405	0.898	0.040 (0.031, 0.049)
CURB-65 + ABG lactate	0.766 (0.717, 0.814)	0.649	0.783	0.466	0.884	0.018(0.009, 0.027)
CURB-65 + LDH	0.784 (0.739, 0.829)	0.777	0.657	0.399	0.910	0.121(0.112, 0.131)
CURB-65 + AST	0.777 (0.728, 0.826)	0.718	0.758	0.464	0.901	0.058(0.049, 0.068)
CURB-65 + NLR	0.791 (0.747, 0.834)	0.812	0.662	0.413	0.923	0.094(0.085, 0.103)
CURB-65 + NLR + FPG	0.802 (0.759, 0.845)	0.914	0.583	0.390	0.958	0.116(0.107, 0.125)
CURB-65 + NLR + ABG lactate	0.797 (0.754, 0.840)	0.846	0.635	0.404	0.934	0.109(0.100, 0.118)
CURB-65 + NLR + LDH	0.805 (0.763, 0.848)	0.745	0.743	0.458	0.908	0.206(0.196, 0.216)
CURB-65 + NLR + AST	0.806 (0.764, 0.849)	0.658	0.827	0.527	0.892	0.159(0.150, 0.169)
CURB-65 + NLR + AST +FPG	0.816 (0.774, 0.858)	0.855	0.633	0.405	0.937	0.181(0.171, 0.190)
CURB-65 + NLR + AST + LDH	0.818 (0.778, 0.859)	0.821	0.710	0.453	0.931	0.247(0.237, 0.258)
CURB-65 + NLR + AST + LDH + FPG	0.824 (0.783, 0.865)	0.880	0.640	0.417	0.948	0.265(0.255, 0.275)
CURB-65 + NLR + ABG lactate + LDH + FPG	0.816 (0.775, 0.857)	0.761	0.755	0.476	0.915	0.235(0.224, 0.245)
CURB-65 + NLR + ABG lactate + AST + LDH + FPG	0.825 (0.785, 0.865)	0.812	0.717	0.457	0.928	0.272(0.262, 0.283)

Extended CURB-65 Scoring: Score 1: CURB-65 + NLR + FPG; Score2: CURB-65 + NLR + ABG lactate + LDH + FPG. ABG, arterial blood gas; AUC, area under the curve; ALT, alanine transaminase; AST, aspartate transaminase; CI, confidence interval; FPG, fasting plasma glucose; IDI, integrated discrimination index; LDH, lactate dehydrogenase; NPV, negative predictive value; NLR, neutrophil to lymphocyte ratio; PPV, positive predictive value.

Table 4 ROC analysis for the Extended CURB-65 Scores predicting in-hospital mortality in low-risk CURB-65 patients.

Extended CURB-65 Scores	AUC	95% CI	Cutoff	Sensitivity (%)	Specificity (%)	PPV (%)	NPV (%)	OR	Mortality
Score 1	0.802	(0.759, 0.845)	2.00	0.914	0.583	0.390	0.958	1.386	1.54%
Score 2	0.816	(0.775, 0.857)	4.00	0.761	0.755	0.476	0.915	1.490	6.96%

AUC, area under the curve; CI, confidence interval; NPV, negative predictive value; PPV, positive predictive value.

Table 5 Extended CURB-65 Score 1 with mortality risk as per risk category.

Parameter	Threshold	Points
Mental status	Confusion	+1
Urea (mg/L)	≥ 7.0 mg/L	+1
Respiratory rate (breaths/min)	≥ 30 breaths/min	+1
Blood pressure (mmHg)	Systolic ≤ 90 mmHg or Diastolic < 60 mmHg	+1
Age (years)	>65	+1
NLR	≥ 4.35	+1
FPG	≥ 9.30	+1
**Aggregate Score 1**	**Severity grade**	**Mortality risk**
< 2.00	Low-risk	1.54%
≥ 2.00	High-risk	21.08%

FPG, fasting plasma glucose; NLR, neutrophil to lymphocyte ratio.

Table 6 Extended CURB-65 Score 2 with mortality risk as per risk category.

**Parameter**	**Threshold**	**Points**
Mental status	Confusion	+1
Urea (mg/L)	≥ 7.0 mg/L	+1
Respiratory rate (breaths/min)	≥ 30 breaths/min	+1
Blood pressure (mmHg)	Systolic ≤ 90 mmHg or Diastolic < 60 mmHg	+1
Age (years)	>65	+1
NLR	≥ 4.35	+1
ABG Lactate	≥ 2.3	+1
LDH	≥ 449.0	+1
FPG	≥ 9.30	+1
**Aggregate Score 2**	**Severity grade**	**Mortality risk**
< 4.00	Low-risk	6.96%
≥ 4.00	High-risk	15.67%

ABG, arterial blood gas; FPG, fasting plasma glucose; LDH, lactate dehydrogenase; NLR, neutrophil to lymphocyte ratio.

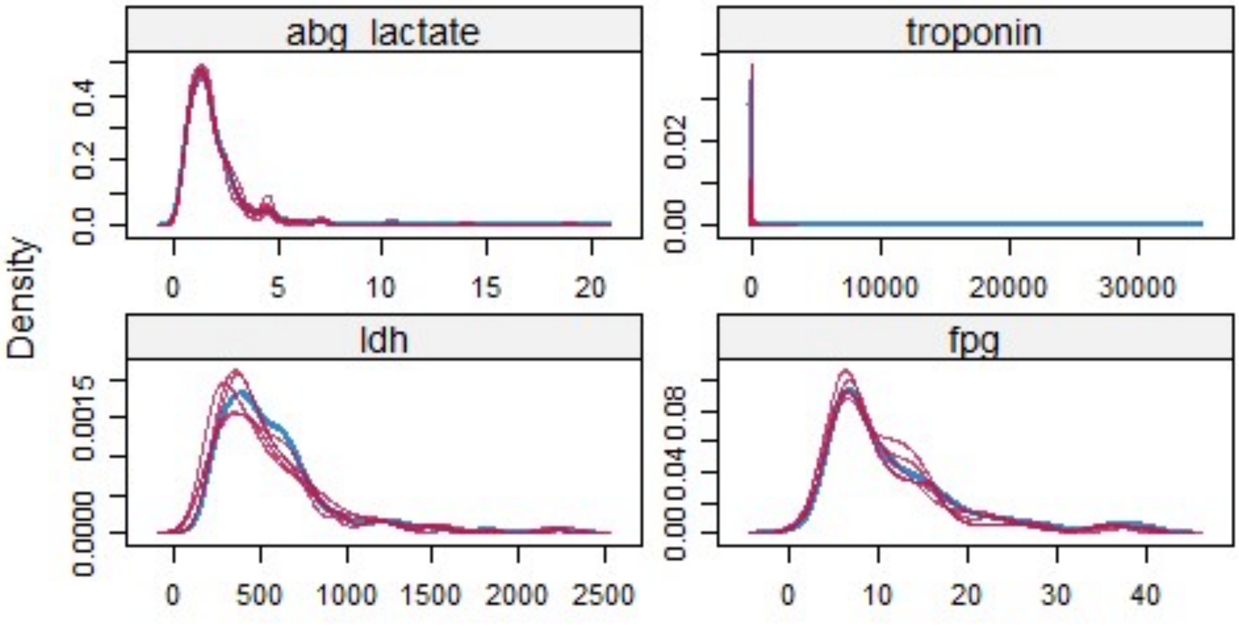

Figure 1 Density plot comparing the distribution of observed (blue line) and imputed values (red lines).

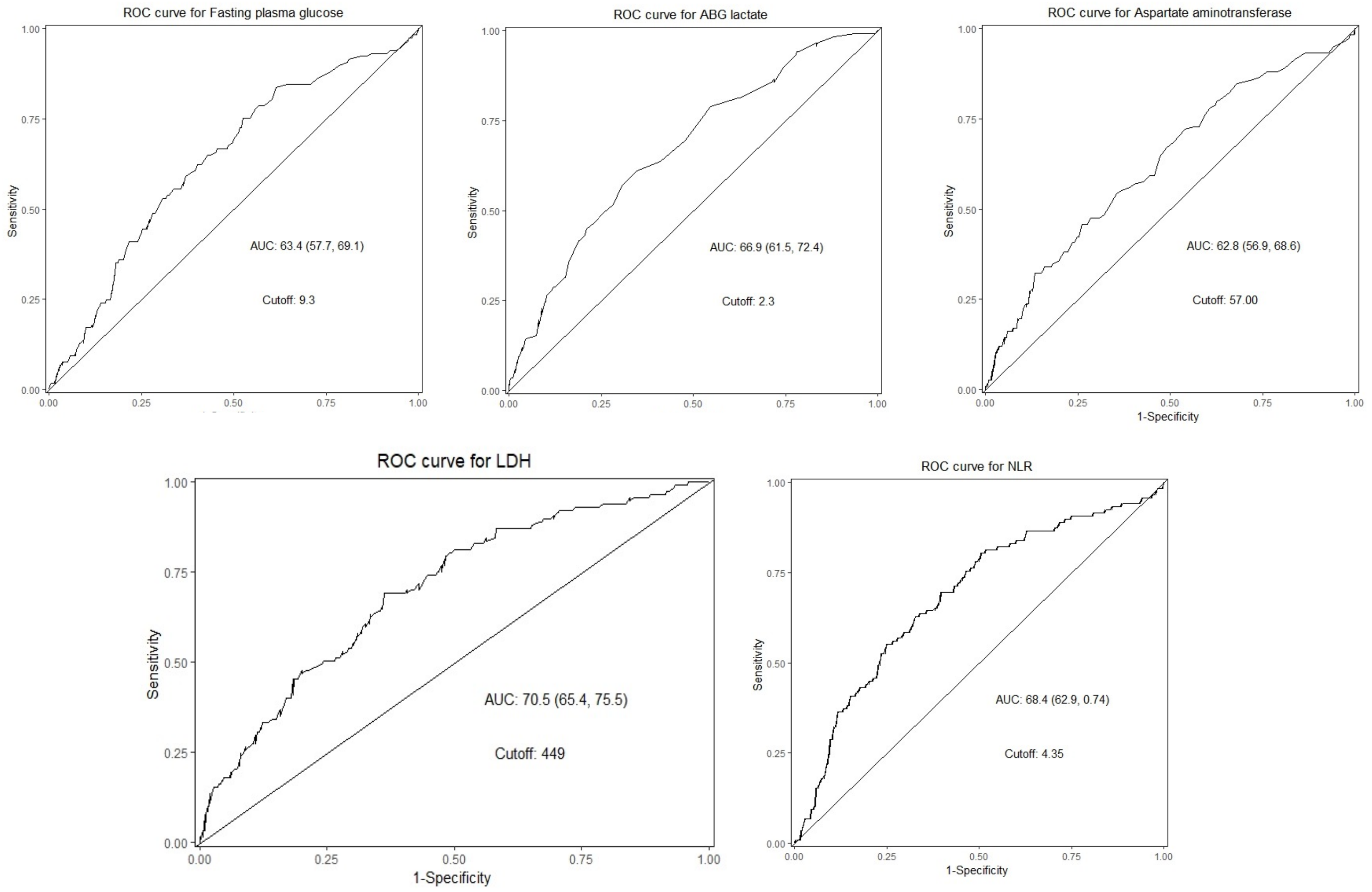

Figure 2 ROC analysis for FPG, lactate on ABG, AST, LDH, NLR for COVID-19 patients. ABG, arterial blood gas; AST, aspartate transaminase; FPG, fasting plasma glucose; LDH, lactate dehydrogenase; NLR, neutrophil to lymphocyte ratio.

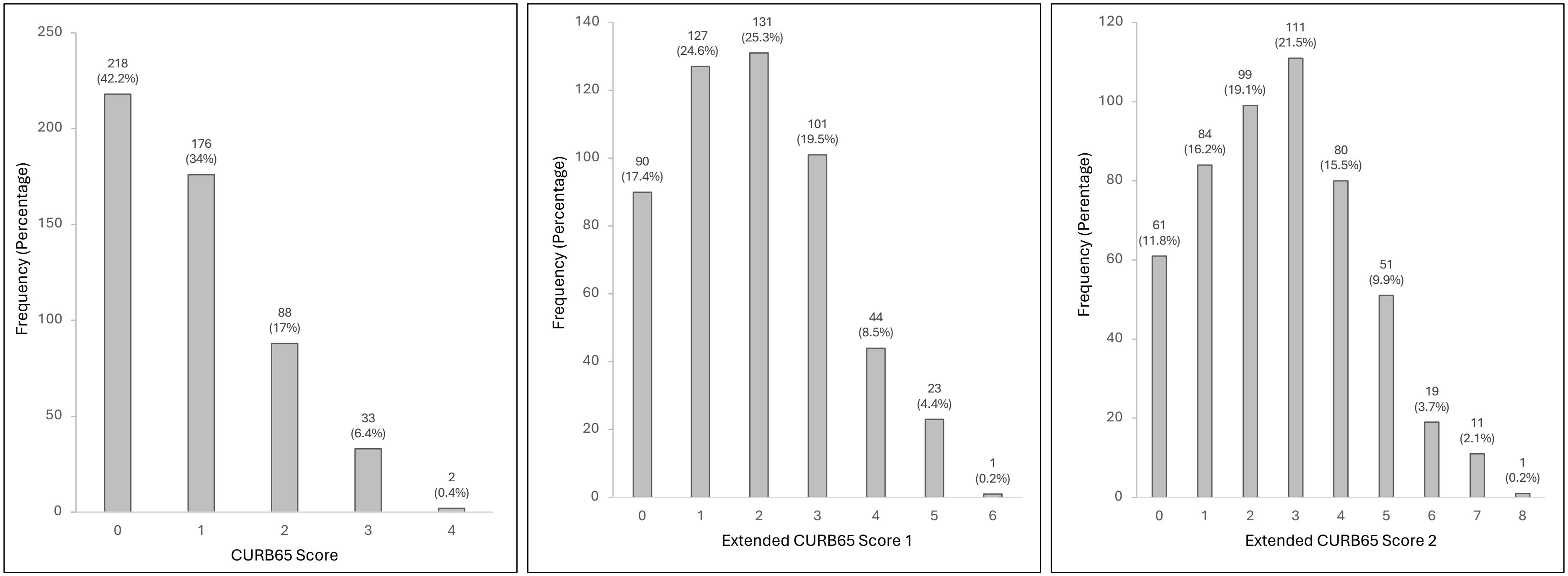

Figure 3 The distribution of patients in the overall cohort (n=517) by CURB-65 and Extended CURB-65 categories.

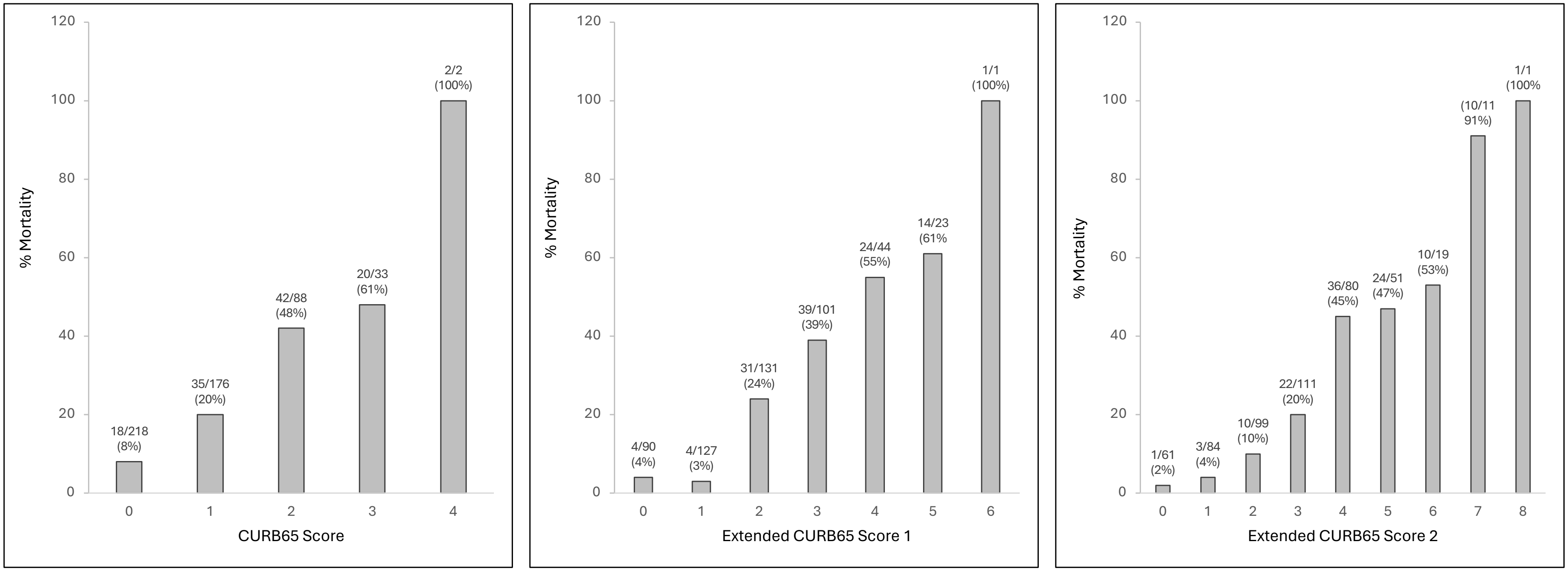

Figure 4 The distribution of mortality in each risk category of CURB-65 and Extended CURB-65 Scores.

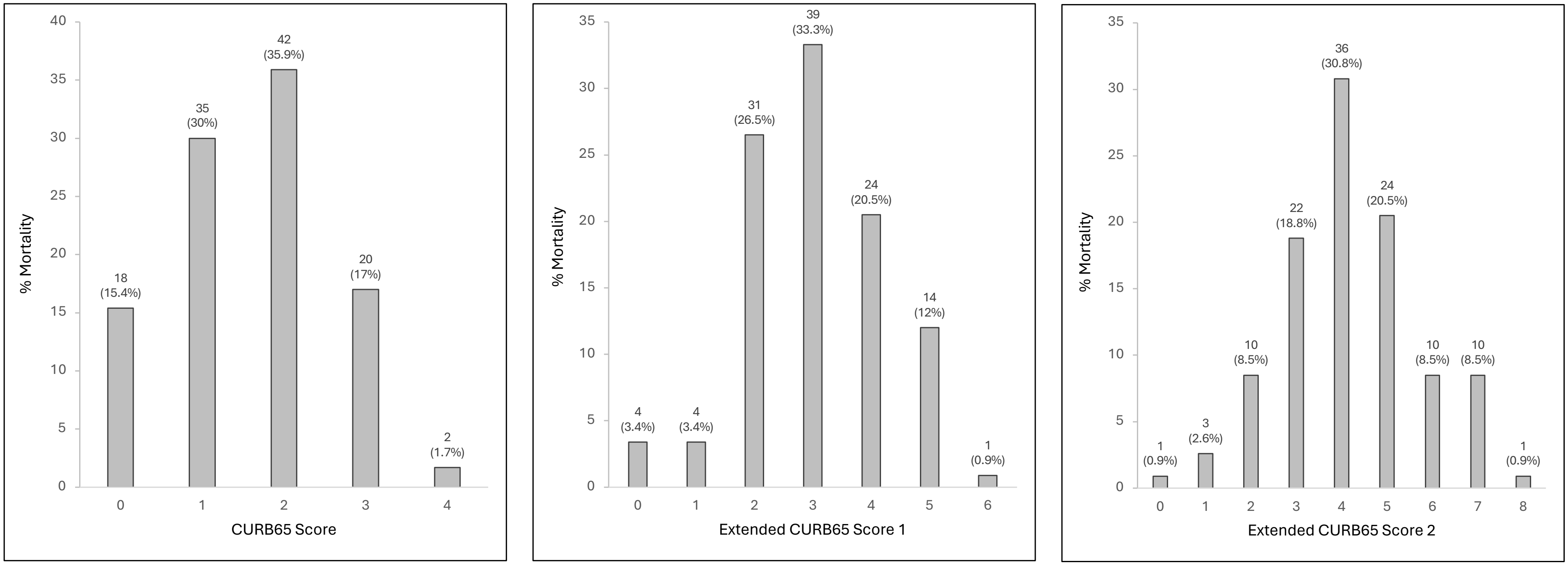

Figure 5 The distribution of mortality in the overall non-survival cohort (n=117) by CURB-65 and Extended CURB-65 categories.
